# Ab Initio Potential Energy Surface and Vibration–Rotation Energy Levels of Aluminum Monohydroxide

**DOI:** 10.1021/acs.jpca.3c05635

**Published:** 2023-10-04

**Authors:** Jacek Koput

**Affiliations:** Department of Chemistry, Adam Mickiewicz University, 61-614 Poznań, Poland

## Abstract

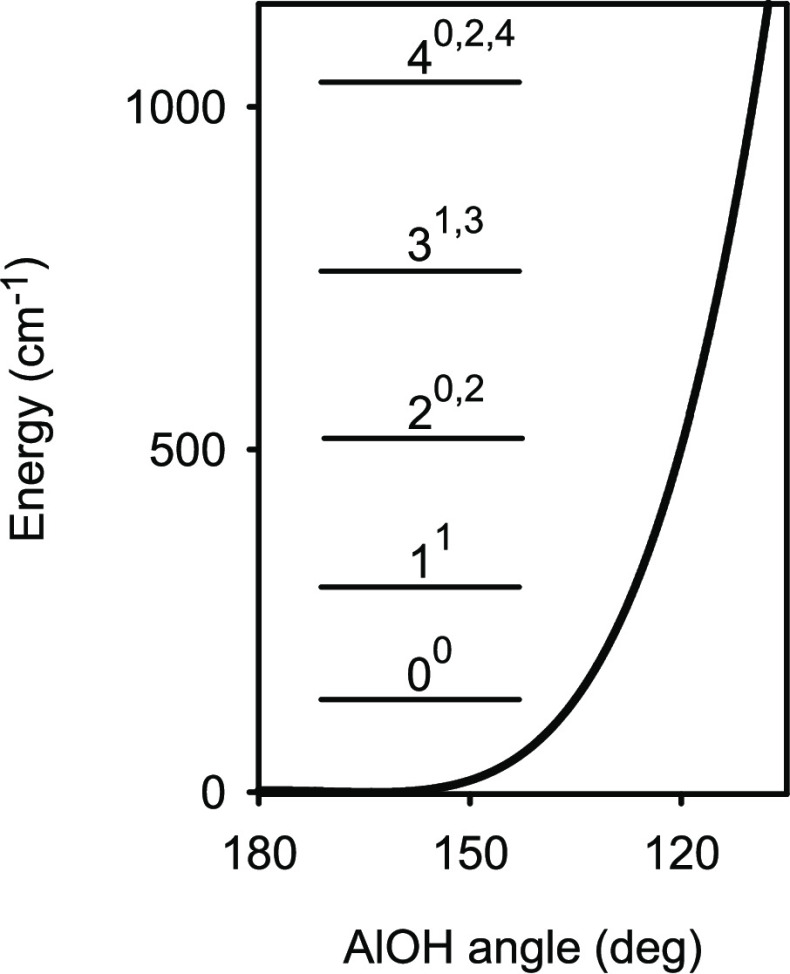

The potential energy surface and vibration–rotation energy levels of aluminum monohydroxide in the *X̃*^1^A′ electronic state have been determined from ab initio calculations. The equilibrium configuration of the AlOH molecule was found to be bent, although with a wide AlOH angle of 163° and a small barrier to linearity of just 4 cm^–1^. The AlOH molecule was definitely confirmed to be quasilinear. The predicted spectroscopic constants of the AlOH, AlOD, ^26^AlOH, and Al^18^OH isotopologues can be useful in a future analysis of high-resolution vibration–rotation spectra of these species.

## Introduction

Aluminum monohydroxide, AlOH, was first reported by Hauge et al.^1^ to be formed by the photolysis of aluminum–water reaction products, largely HAlOH. The low-resolution infrared spectra of AlOH, Al^18^OH, and AlOD in an argon matrix at 15 K were observed and the vibrational fundamental wavenumber ν_1_ of the AlO stretching mode was determined to be 810.3, 785.2, and 795.2 cm^–1^, respectively. The vibrational fundamental wavenumber ν_3_ for the OH stretching mode of AlOH was determined to be 3790 cm^–1^. The electronic spectra of AlOH and AlOD were observed by Pilgrim et al.^2^ From an analysis of the vibrational progressions, the vibrational energy difference Δ*G*_1/2_ for the AlO stretching mode of AlOH was derived to be 895 cm^–1^. The rotational spectra of AlOH and AlOD were observed by Apponi et al.,^3^ and the spectroscopic constants for the ground vibrational state were derived. The infrared spectra of AlOH and AlOD in an argon matrix at 10 K were also observed by Wang and Andrews.^4^ The fundamental wavenumbers ν_1_ and ν_3_ of AlOH were determined to be 810.4 and 3787.0 cm^–1^, respectively. For AlOD, the corresponding values were determined to be 794.7 and 2799.8 cm^–1^, respectively.

Following the theoretical study by Vacek et al.,^5^ the structure of the AlOH molecule was described in all of the above-mentioned experimental studies^1−4^ as quasilinear. The equilibrium configuration of a quasilinear molecule is bent. However, the bending potential energy function is flat near a minimum, with a sizable barrier to linearity. For the barrier height smaller than the bending fundamental wavenumber, the pattern of vibration–rotation energy levels of a quasilinear molecule is irregular, and it resembles that of a highly nonrigid linear molecule. To our knowledge, vibration–rotation transitions between the bending energy levels of AlOH were not observed experimentally so far, and therefore, the quasilinearity of the AlOH molecule was never verified.

Vacek et al.^5^ investigated the structure and AlOH–HAlO isomerization at various levels of theory up to CCSD(T)/TZP. The equilibrium structure of AlOH was predicted to be bent, with an equilibrium AlOH angle of 162.6° and a barrier to linearity smaller than 1 kcal/mol. Similar results were obtained by Hirota et al.,^6^ with the barrier height estimated to be just 2 cm^–1^ at the MP3 and CISD/6-311G(2df,2pd) levels of theory. Li et al.^7^ investigated the three lowest-lying singlet electronic states of AlOH at various levels of theory up to CC3/aug-cc-pVQZ. Using the coupled-cluster methods with a partial iterative treatment of connected triple excitations, CCSDT-3 and CC3, the equilibrium AlOH angle and the barrier to linearity for the ground electronic state were determined to be 156.8° and 14 cm^–1^, respectively. Li et al.^7^ concluded that the equilibrium AlOH angle appeared to be quite sensitive to both the treatment of correlation effects and the size of the one-particle basis set. The neutral, cationic, and anionic forms of AlOH and HAlO were characterized by Sikorska and Skurski^8^ at the QCISD/aug-cc-pVTZ level of theory, confirming quasilinearity of the neutral AlOH species. The structure and energetics of AlOH and HAlO were investigated by Trabelsi and Francisco^9^ using the conventional and explicitly correlated (F12) methods; the single-reference coupled-cluster CCSD(T) and multireference configuration interaction (MRCI) approaches were applied, all in conjunction with the aug-cc-pV5Z basis set. Using the single-reference coupled-cluster methods, CCSD(T) and CCSD(T)-F12, the equilibrium AlOH angle was predicted to be 160.9 and 161.0°, respectively. Using the multireference configuration interaction methods, MRCI+Q and MRCI-F12, the equilibrium AlOH angle was predicted to be 139.0 and 134.2°, respectively. Such a large difference between the single- and multireference approaches indicates that the higher-order electron correlation effects may be important for accurately describing the ground electronic state of AlOH. Somewhat surprisingly, the harmonic frequency of the AlOH bending mode ν_2_ was found^9^ to be still harder to predict. Using the CCSD(T), CCSD(T)-F12, MRCI+Q, and MRCI-F12 methods, the ω_2_ value was calculated to be 133.3, 129.4, 380.0, and 485.9 cm^–1^, respectively. Concerning the barrier to linearity of AlOH, Trabelsi and Francisco^9^ reported only the value of 5.3 cm^–1^ obtained using the CCSD(T)-F12 method. Handy et al.^10^ investigated the three-dimensional potential energy surface of AlOH at the CCSD(T)/cc-pVQZ level of theory. The vibration–rotation energy levels of the main isotopologue AlOH were calculated using the variational method, and the vibrational fundamental wavenumbers ν_1_, ν_2_, and ν_3_ were predicted to be 829, 145, and 3838 cm^–1^, respectively. Fortenberry et al.^11^ determined the quartic force field (QFF) of AlOH both at the CCSD(T)-F12/cc-pVTZ-F12 level of theory (F12-TZ) and by using the so-called CcCR composite approach (see refs (12,13) for further details of the CcCR QFF approach). The vibration–rotation energy levels and the related spectroscopic constants were then calculated by using the second-order perturbational method. Both of the QFF approaches were considered^11^ “to provide exceptional accuracy” in predicting vibrational–rotational spectroscopic constants. In particular, using the F12-TZ and CcCR QFF approaches, the equilibrium AlOH angle was predicted to be 156.0 and 159.7°, respectively. Using the F12-TZ QFF approach, the vibrational fundamental wavenumbers ν_1_, ν_2_, and ν_3_ for the main isotopologue AlOH were predicted to be 813.6, 177.3, and 3808.5 cm^–1^, respectively. Using the CcCR QFF approach, the corresponding wavenumbers were predicted to be 817.7, 146.7, and 3816.8 cm^–1^, respectively. The molecular parameters of AlOH predicted using both QFF approaches, especially those related to the AlOH bending mode ν_2_, differ significantly. Moreover, Fortenberry et al.^11^ stated that “in the reported ν_2_ frequencies for both QFFs, the cubic and quartic terms have been removed from inclusion in the VPT2 computations. Retaining them leads to egregious positive anharmonicities that are almost certainly faulty.”

Despite the high level of theory used in previous studies,^5−11^ the molecular parameters of AlOH were predicted to be significantly different. The aim of this work is to provide the accurate state-of-the-art potential energy surface for the ground electronic state of AlOH and to discuss the effects which should be taken into account in order to predict the vibration–rotation energy levels of AlOH to near “spectroscopic” accuracy. In computational chemistry, such an accuracy means error bars on the predicted vibrational fundamental wavenumbers smaller than ±1 cm^–1^ and those on the predicted equilibrium internuclear distances smaller than ±0.0001 Å.

## Method of Calculation

Calculations of the molecular parameters of AlOH closely follow those reported recently for magnesium monohydroxide.^14^ The electronic energy of AlOH was determined using the conventional coupled-cluster method including single and double excitations and a perturbational correction due to connected triple excitations, CCSD(T).^15−18^ The one-particle basis sets employed were the correlation-consistent valence basis sets up to septuple-zeta quality, cc-pV*n*Z (*n* = D–7).^19−24^ The outer-core 2s- and 2p-like orbitals of aluminum were treated as valence, and therefore, the basis sets for aluminum were augmented with tight functions (C). Because the natural charge at the oxygen atom of AlOH was estimated to be – 1.3 *e*, the basis sets for oxygen were augmented with diffuse functions (aug). The total energies of AlOH were thus calculated using the cc-pCV*n*Z, aug-cc-pV*n*Z, and cc-pV*n*Z basis sets for aluminum, oxygen, and hydrogen, respectively. These basis sets are further referred to as “*n*Z”. Accordingly, the extended valence active space was used in the correlation treatment. This active space thus included all but aluminum and oxygen 1s electrons. Calculations were performed with the MOLPRO package of ab initio programs^25^ unless otherwise noted.

Vibration–rotation energy levels of AlOH were determined using the variational method, the RVIB3 program.^26,27^ The six-dimensional vibration–rotation Hamiltonian consists of an exact representation of the kinetic energy operator and an approximate representation of the potential energy operator, both defined in terms of the internal valence coordinates. The vibration–rotation wave function is a linear combination of products of the vibrational contracted functions and the rotational symmetric-top functions. The number of contracted two-dimensional stretching functions was 49 and the number of contracted bending functions was 22, leading to a total of 1078 vibrational basis set functions. The energy levels were calculated using the nuclear masses of aluminum, oxygen, and hydrogen.

## Results and Discussion

To determine the shape of the potential energy surface of AlOH, the total energies were calculated at the CCSD(T)/*n*Z (*n* = 5 and 6) level of theory, with the extended valence active space at 203 symmetry unique points. The AlO and OH bond lengths were sampled in the range of 1.3–2.3 and 0.7–1.4 Å, respectively. The AlOH valence angle was sampled in the range of 180–60°. For the largest basis set, 7Z, the total energies of AlOH were calculated only for a limited number of points in the vicinity of the equilibrium and linear configurations. In these calculations, only the structural parameters for the equilibrium and linear configurations of AlOH were obtained.

The potential energy surfaces were approximated by a three-dimensional (3D) expansion along the internal valence coordinates. The Simons–Parr–Finlan coordinates^28^ were chosen for the AlO and OH stretching modes. These coordinates are termed *q*_1_ and *q*_2_, respectively. The curvilinear displacement coordinate^29^ was chosen for the AlOH bending mode. This coordinate is defined as the supplement of the AlOH valence angle and is termed as θ: θ = π – ∠(AlOH). The potential energy surface of AlOH is written as the polynomial expansion
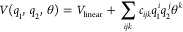
1where *V*_linear_ is the total energy at the linear configuration of the AlOH molecule, and the index *k* takes only even values. The linear configuration was taken as a reference configuration. The expansion coefficients *c*_*ijk*_ were obtained from a least-squares fit of eq 1 to all of the computed total energies of AlOH, and 39 coefficients were statistically significant. The root-mean-square (rms) deviations of the fits were about 1.3 μ*E*_h_ (0.3 cm^–1^).

The potential energy surfaces were used to calculate the vibration–rotation energy levels of the main isotopologue AlOH, with the rotational quantum number *N* ranging from 0 to 7. The obtained molecular parameters are listed in Table 1. The parameters quoted include the structural parameters for the equilibrium and linear configurations, the total energy at a minimum, the barrier to linearity, the vibrational fundamental wavenumbers ν_*i*_ (*i* = 1–3), and the ground-state effective rotational constant *B*_(0,0°,0)_. The equilibrium configuration of AlOH in the electronic *X̃*^1^A′ state was found to be bent, thus confirming the results of the previous theoretical studies.^5−11^ At the highest level of theory applied in this work, CCSD(T)/7Z, the equilibrium AlOH angle, was predicted to be 161.85°. The corresponding height of the barrier to linearity of the AlOH molecule was calculated to be just 4.7 cm^–1^. Because the ground state of the AlOH bending mode ν_2_ is located well above the barrier top (see below), the AlOH molecule is effectively linear. Therefore, it is most adequate to describe the vibration–rotation energy levels of AlOH in terms of the linear molecule model. For the AlO and OH stretching modes, the energy levels are labeled with quantum numbers ν_1_ and ν_3_, respectively. For the doubly degenerate AlOH bending mode, the two quantum numbers ν_2_ and *l*_2_ (*l*_2_ ≡ *l* for abbreviation) are used. For a given vibrational state (ν_1_, ν_2_^*l*^, ν_3_), the effective rotational constant *B*_(ν_1_,ν_2_^*l*^,ν_3_)_ and the quartic centrifugal distortion constant *D*_(ν_1_,ν_2_^*l*^,ν_3_)_ were determined by least-squares fitting the predicted rotational transition energies with a power series in [*N*(*N* + 1) – *l*^2^]. As shown in Table 1, the total energy of AlOH at the CCSD(T)/7Z level of theory is converged to better than 4 m*E*_h_. The changes in the predicted structural parameters *r*(AlO), *r*(OH), and ∠(AlOH) beyond the 7Z basis set are expected to be smaller than 0.0003, 0.00001, and 0.1°, respectively. The height of the barrier to linearity of the AlOH molecule is expected to be accurate to ±1 cm^–1^. The best predicted values of the vibrational fundamental wavenumbers ν_*i*_ and the effective rotational constant *B*_(0,0°,0)_ of AlOH are expected to be accurate to ±1 cm^–1^ and ±5 MHz, respectively.

**Table 1:** Molecular Parameters for the *X̃*^1^A′ State of AlOH Determined at the CCSD(T)/*n*Z Level of Theory

	*n* = 5	*n* = 6	*n* = 7
Equilibrium Configuration			
*r*(AlO) (Å)	1.67382	1.67324	1.67332
*r*(OH) (Å)	0.94983	0.94983	0.94988
∠(AlOH) (deg)	162.82	162.57	161.85
*E*+318^a^ (hartree)	–0.142172	–0.149579	–0.153203
Linear Configuration			
*r*(AlO) (Å)	1.66950	1.66878	1.66849
*r*(OH) (Å)	0.94908	0.94903	0.94902
Δ*E*^b^ (cm^–1^)	3.8	3.9	4.7
			
ν_1_^c^ (cm^–1^)	827.7	828.9	829.1^e^
ν_2_ (cm^–1^)	158.4	158.1	158.1
ν_3_ (cm^–1^)	3816.7	3816.6	3816.7
*B*_(0,0°,0)_^d^ (MHz)	15,709.0	15,722.4	15,727.5^e^

a The total energy at a minimum.

b The barrier to linearity.

c The vibrational fundamental wavenumbers.

d The ground-state effective rotational constant.

e Calculated using the *n* = 7 structural parameters and the *n* = 6 anharmonic force field.

The best estimate of the potential energy surface of AlOH [CCSD(T)/6Z], in conjunction with the best estimate of the structural parameters [CCSD(T)/7Z], was used in further calculations. It was then corrected gradually for the inner-core–electron correlation, higher-order electron correlation, scalar relativistic effects, and adiabatic effects. The energy corrections were calculated at each of the 203 symmetry unique points mentioned above.

The inner-core–electron correlation effects were calculated as differences in the total energy of AlOH obtained by using the CCSD(T)/5Z method with the two active spaces. The first active space included all but aluminum 1s electrons, whereas the second active space was the extended valence space described above. Only a contribution of oxygen 1s electrons is considered in this study. Thus, the basis set for oxygen was augmented with tight functions. The correlation effects of inner-core electrons of aluminum are likely to be negligible because of the huge orbital energy gap. In the vicinity of the equilibrium configuration of AlOH, the total energy contribution due to this effect amounts to −61 m*E*_h_.

The effects of electron correlation beyond the CCSD(T) level of approximation were estimated from calculations using the CCSDT and CCSDTQ methods, both with the extended valence active space. The calculations were performed using the CFOUR program.^30^ The higher-order electron correlation correction to the total energy of AlOH is composed of a sum of two terms. The first term is a difference, *E*[CCSDT/QZ] – *E*[CCSD(T)/QZ], where *E*[···] denotes the total energy at a given level of theory. The second term is a difference of *E*[CCSDTQ/DZ] – *E*[CCSDT/DZ]. In the vicinity of the equilibrium configuration of AlOH, the first term was calculated to be −50 μ*E*_h_, whereas the second term was calculated to be −700 μ*E*_h_. For comparison, a contribution to the correlation energy of AlOH due to connected triple excitations was calculated to be about 17,000 μ*E*_h_ at the CCSDT/QZ level of theory. Thus, the difference between the iterative and perturbational treatments of connected triple excitations is almost negligible for AlOH in its ground electronic state. The higher-order electron correlation correction to the total energy of AlOH is dominated by the iterative contribution due to connected quadruple excitations.

The scalar relativistic effects were estimated using the exact-2-component (X2C) approach.^31^ This correction was determined as a difference in the total energy of AlOH calculated using either the X2C or nonrelativistic Hamiltonian, both energies obtained at the CCSD(T)/5Z (uncontracted) level of theory. In the vicinity of the equilibrium configuration of AlOH, it amounts to about −489 m*E*_h_.

The adiabatic effects were estimated by calculating the diagonal Born–Oppenheimer correction (DBOC) at the CCSD/TZ level of theory.^32^ The calculations were performed using the CFOUR program.^30^ In the vicinity of the equilibrium configuration of the AlOH and AlOD isotopologues, the DBOC was predicted to be about 6.86 and 6.76 m*E*_h_, respectively.

**Figure 1:**
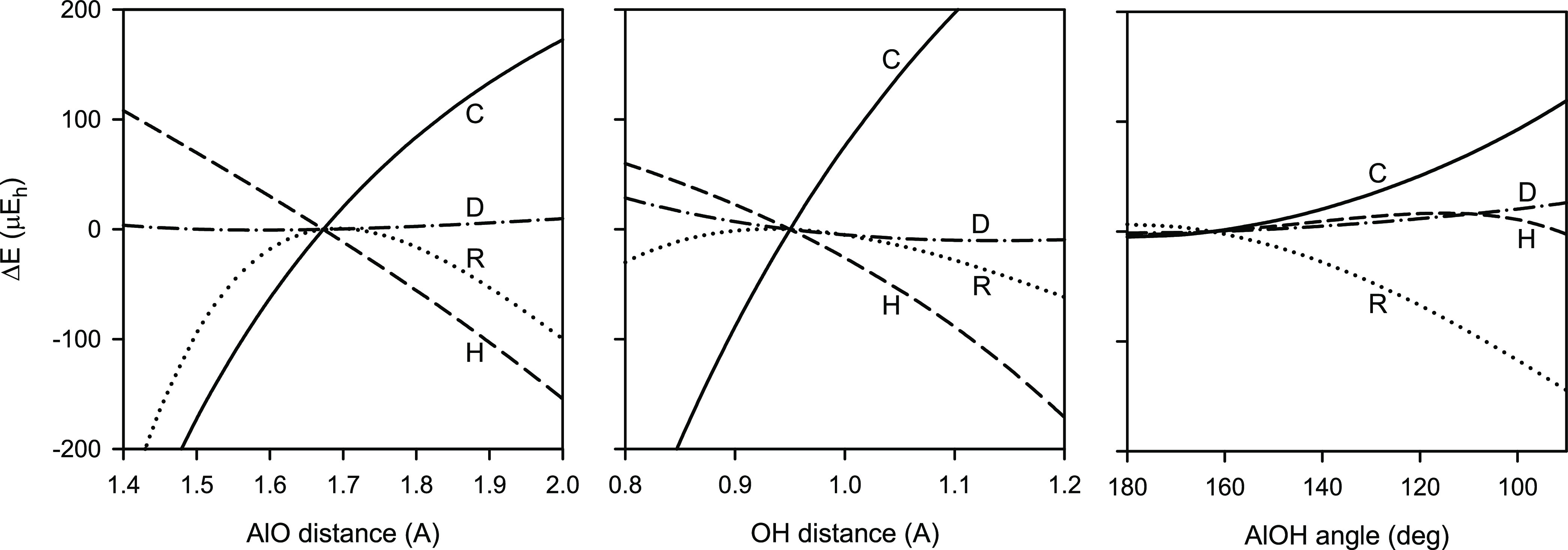
Changes in the total energy Δ*E* for the *X̃*^1^A′ state of AlOH due to the inner-core–electron correlation (C, solid lines), higher-order electron correlation (H, dashed lines), scalar relativistic (R, dotted lines), and adiabatic (D, dashed-dotted lines) effects as functions of the internal valence coordinates. The relative values of Δ*E* are plotted with the absolute changes Δ*E* taken as the origin of the Δ*E* axis. These reference values were calculated for the equilibrium configuration of AlOH with *r*(AlO) = 1.672 Å, *r*(OH) = 0.949 Å, and ∠(AlOH) = 162.7°. For each one-dimensional plot, the other two internal valence coordinates were kept fixed at the equilibrium values.

Figure 1 illustrates changes in the total energy for the *X̃*^1^A′ state of AlOH due to the inner-core–electron correlation (C), higher-order electron correlation (H), scalar relativistic (R), and adiabatic (D) effects along the internal valence coordinates. As in the case of the similar molecules, MgOH and BeOH,^14,33^ the changes along the internuclear distances *r*(AlO) and *r*(OH) were found to be similar and qualitatively different than that along the valence angle ∠(AlOH).

The molecular parameters of the main isotopologue AlOH, calculated with the potential energy surfaces gradually corrected for the effects mentioned above, are given in Table 2. The most interesting changes are observed for descriptors of the molecular structure and vibration–rotation dynamics of AlOH, namely, the ground-state effective rotational constant and the vibrational fundamental wavenumbers. Upon accounting for the C, H, and R effects, the constant *B*_(0,0°,0)_ changes by 16.2, −8.2, and 0.8 MHz, respectively. The total correction C+H+R thus amounts to only 8.8 MHz, being just about twice larger than the estimated uncertainty in the constant *B*_(0,0°,0)_ due to the limited size of the one-particle basis set. For the fundamental wavenumber ν_1_, the corrections C, H, and R are predicted to be 1.3, −1.2, and −1.7 cm^–1^, respectively. For the fundamental wavenumber ν_2_, the corrections C, H, and R are predicted to be 4.1, −0.2, and −2.4 cm^–1^, respectively. And for the fundamental wavenumber ν_3_, the corrections C, H, and R are predicted to be 6.9, −5.3, and −3.3 cm^–1^, respectively. The total corrections C+H+R to the vibrational fundamental wavenumbers ν_1_, ν_2_, and ν_3_ amount thus to only −1.6, 1.5, and −1.7 cm^–1^, respectively. The changes predicted for the molecular parameters of AlOH are consistent with the changes in the total energy of AlOH along the internal valence coordinates, as shown in Figure 1, and tend to partly cancel each other out. The best predicted Born–Oppenheimer molecular parameters for the *X̃*^1^A′ state of AlOH are listed in the column headed “V+C+H+R”, and the corresponding potential energy surface is given in Table S1 of the Supporting Information. The best predicted adiabatic molecular parameters for the main isotopologue AlOH are listed in the rightmost column of Table 2. The adiabatic corrections appeared to be small, being just 0.2 MHz for the rotational constant *B*_(0,0°,0)_ and about 0.3 cm^–1^ on average for the vibrational fundamental wavenumbers. Note that corrections C, H, R, and D for the barrier to linearity of AlOH were predicted to be also small, being only −2.0, – 0.1, 1.2, and −0.2 cm^–1^, respectively. The total correction C+H+R to the barrier of linearity of the AlOH molecule is predicted to be just −0.9 cm^–1^, being essentially equal to the estimated uncertainty in the calculated barrier height.

**Table 2:** Molecular Parameters^a^ for the *X̃*^1^A′ State of AlOH Determined Using Various Potential Energy Surfaces

	V^b^	V+C^c^	V+C+H^c^	V+C+H+R^c^	V+C+H+R+D^c^
Equilibrium Configuration					
*r*(AlO) (Å)	1.67332	1.67145	1.67178	1.67243	1.67228
*r*(OH) (Å)	0.94988	0.94887	0.94910	0.94925	0.94929
∠(AlOH) (deg)	161.85	164.13	164.30	162.73	163.02
*E*+318 (hartree)	–0.153203	–0.214651	–0.215433	–0.704799	–0.697941
Linear Configuration					
*r*(AlO) (Å)	1.66849	1.66774	1.66815	1.66807	1.66806
*r*(OH) (Å)	0.94902	0.94822	0.94845	0.94847	0.94853
Δ*E* (cm^–1^)	4.7	2.7	2.6	3.8	3.6
					
ν_1_ (cm^–1^)	829.1	830.4	829.2	827.5	827.8
ν_2_ (cm^–1^)	158.1	162.2	162.0	159.6	160.0
ν_3_ (cm^–1^)	3816.7	3823.6	3818.3	3815.0	3815.2
*B*_(0,0°,0)_ (MHz)	15,727.5	15,743.7	15,735.5	15,736.3	15,736.5

a See Table 1.

b The best estimates determined using the CCSD(T) method with the extended valence active space (see text).

c Including additional corrections for the inner-core–electron correlation (C), higher-order electron correlation (H), scalar relativistic (R), and adiabatic (D) effects.

**Figure 2:**
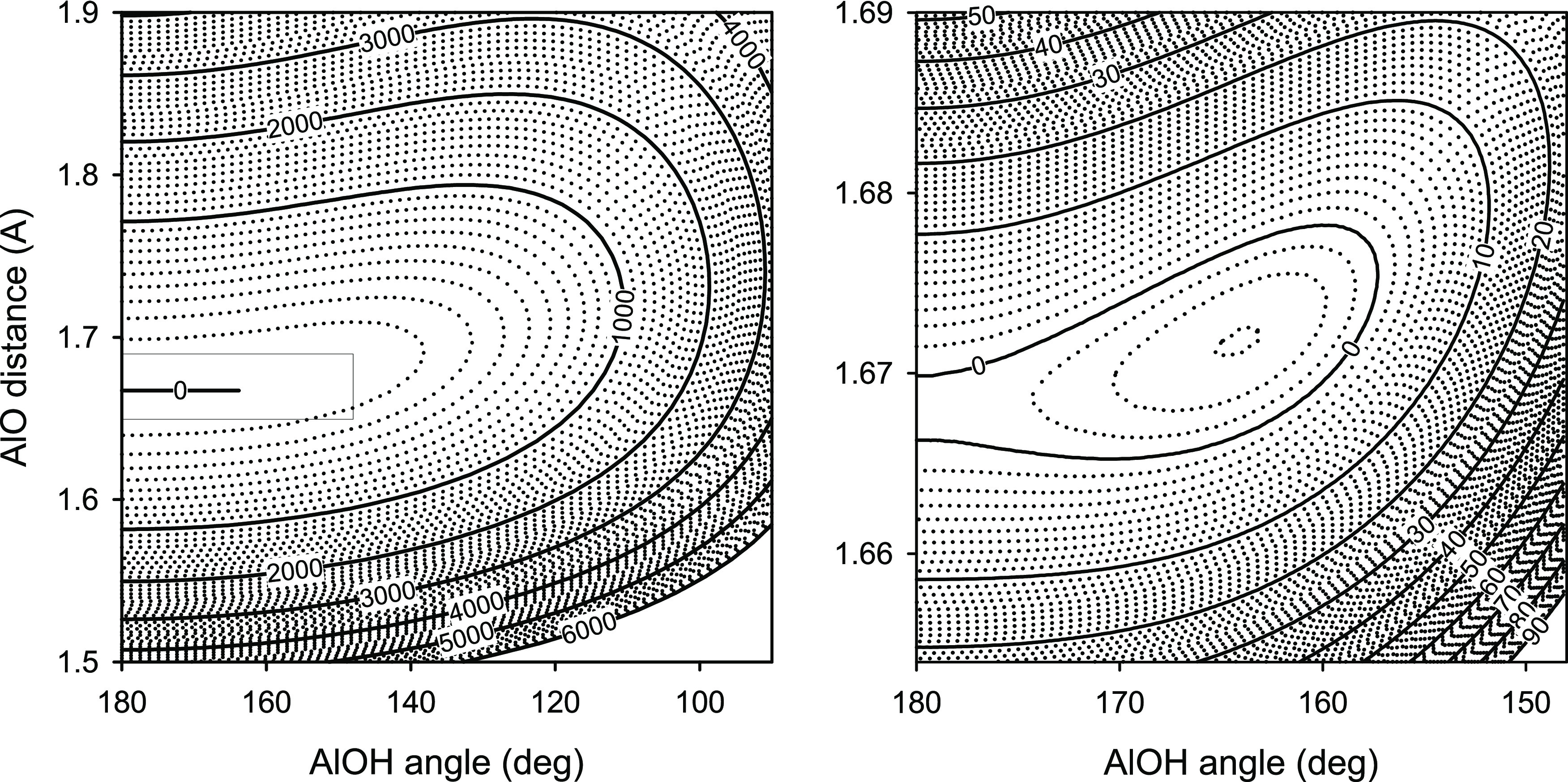
Contour plots showing two-dimensional slices through the adiabatic potential energy surface of AlOH along the internuclear distance *r*(AlO) and valence angle ∠(AlOH), calculated for *r*(OH) = 0.949 Å. The right-hand panel is an expanded view of the rectangular area around the minimum marked in the left-hand panel. Energy contours (in cm^–1^) are plotted relative to the reference linear configuration of AlOH. The minor contours are plotted in the left- and right-hand panels every 100 and 1 cm^–1^, respectively.

Figure 2 shows the contour plot of the predicted adiabatic potential energy surface of AlOH along the internal valence AlO stretching and AlOH bending coordinates. It was found that interactions between pairs of the vibrational modes of AlOH are weak, especially between pairs including the OH stretching mode ν_3_. The right-hand panel illustrates the potential energy surface in the close vicinity of the equilibrium configuration of AlOH. Both the panels show clearly large anharmonicity of the bending mode ν_2_ of the AlOH molecule.

The adiabatic potential energy surfaces were used to determine the vibration–rotation energy levels for the *X̃*^1^A′ state of the AlOH, AlOD, ^26^AlOH, and Al^18^OH isotopologues. The predicted *N* = |*l*| term values are given in Table 3. The energy levels listed include all of the vibrational levels of AlOH with |*l*| ≤ 6, up to about 1600 cm^–1^ above the ground state. To our knowledge, there are no experimental data available to compare with, except for the ν_1_ and ν_3_ vibrational fundamental wavenumbers of AlOH, AlOD, and Al^18^OH determined from an analysis of the low-resolution infrared spectra in an argon matrix.^1,4^ The fundamental wavenumber ν_1_ for the AlOH, AlOD, and Al^18^OH isotopologues was determined to be 810, 795, and 785 cm^–1^, respectively. The fundamental wavenumber ν_3_ for the AlOH and AlOD isotopologues was determined to be 3787 and 2800 cm^–1^, respectively. Considering the possibly sizable matrix shift effect,^34^ the predicted vibrational fundamental wavenumbers are in good agreement with the observed values. In this case, however, these are the experimental isotopic shifts that are most directly comparable to the ab initio predicted counterparts. For the fundamental wavenumber ν_1_, the D- and ^18^O-isotopic shifts were observed^1^ to be −15.1 and −25.1 cm^–1^, respectively. The corresponding values were predicted in this work to be −15.1 and −26.0 cm^–1^, respectively. For the fundamental wavenumber ν_3_, the D-isotopic shift was observed^4^ to be −987.2 cm^–1^, compared to the predicted value of −993.9 cm^–1^. The vibrational fundamental wavenumbers ν_1_, ν_2_, and ν_3_ are predicted in this work for the main isotopologue AlOH to be 827.8, 160.0, and 3815.2 cm^–1^, respectively. These values differ significantly from those predicted by Fortenberry et al.^11^ Using the two F12-TZ/CcCR quartic force field approaches, the corresponding values were calculated to be 813.6/817.7, 177.3/146.7, and 3808.5/3816.8 cm^–1^, respectively. A test of the predictive power of both the approaches applied by Fortenberry et al.^11^ and the approach applied in this study will have to await future high-resolution spectroscopy experiments on AlOH and its isotopologues.

**Table 3:** Predicted Adiabatic *N* = |*l*| Vibration–Rotation Term Values *T*_(ν_1_,ν_2_^*l*^,ν_3_)_ (in cm^–1^) for the *X̃*^1^A′ State of AlOH, AlOD, ^26^AlOH, and Al^18^OH

ν_1_,ν_2_^*l*^,ν_3_	AlOH	AlOD	^26^AlOH	Al^18^OH
0,0°,0^a^	0.	0.	0.	0.
0,1^1^,0	160.0	107.1	160.1	158.7
0,2^2^,0	367.3	244.5	367.4	364.3
0,2°,0	384.6	262.4	384.7	380.9
0,3^3^,0	614.5	406.8	614.6	609.5
0,3^1^,0	627.7	427.3	627.9	621.6
1,0°,0	827.8	812.7	833.8	801.8
0,4^4^,0	897.7	591.0	897.8	890.4
0,4^2^,0	898.2	608.2	898.4	889.5
0,4°,0	899.3	614.4	899.6	890.1
1,1^1^,0	979.1	912.7	985.0	952.5
1,2^2^,0	1179.6	1046.5	1185.5	1151.9
0,5^1^,0	1191.3	812.4	1191.6	1178.8
0,5^3^,0	1197.6	805.8	1197.8	1186.0
1,2°,0	1201.0	1067.8	1206.9	1172.2
0,5^5^,0	1214.6	795.2	1214.7	1204.8
1,3^3^,0	1421.2	1200.9	1427.1	1392.1
1,3^1^,0	1439.0	1222.4	1445.0	1408.4
0,6°,0	1499.6	1023.0	1500.1	1483.4
0,6^2^,0	1506.2	1021.5	1506.6	1490.3
0,6^4^,0	1525.9	1020.2	1526.2	1511.2
0,6^6^,0	1563.6	1018.2	1563.8	1551.1
2,0°,0	1645.0	1615.2	1656.8	1593.9
0,0°,1	3815.2	2821.3	3815.1	3802.7

a The zero-point energy is calculated for AlOH, AlOD, ^26^AlOH, and Al^18^OH to be 2513.3, 1939.3, 2516.4, and 2488.4 cm^–1^, respectively.

The vibrational energy levels of the AlOH, AlOD, ^26^AlOH, and ^18^OH isotopologues were further characterized by the harmonic frequencies ω_*i*_ and anharmonicity constants *x*_*ij*_, *y*_*ijk*_, and *g*_22_, as given in Table 4. Note that as for the bending mode ν_2_ of the MgOH molecule,^14^ the anharmonicity constant *x*_22_ is positive and it amounts to about one-fifth of the harmonic frequency ω_2_.

**Table 4:** Predicted Vibrational Constants ω_*i*_, *x*_*ij*_, *y*_*ijk*_, and *g*_22_ (in cm^–1^) for the *X̃*^1^A′ State of AlOH, AlOD, ^26^AlOH, and Al^18^OH

	AlOH	AlOD	^26^AlOH	Al^18^OH
ω_1_	846.88	826.94	853.32	820.29
ω_2_	120.06	78.10	120.20	119.13
ω_3_	4008.15	2926.87	4008.54	3995.11
*x*_11_	–5.26	–5.13	–5.34	–4.91
*x*_22_	24.34	17.50	24.32	24.00
*x*_33_	–85.30	–45.61	–85.30	–84.69
*x*_12_	–7.40	–2.01	–7.45	–7.02
*x*_13_	–3.25	–3.08	–3.74	–4.12
*x*_23_	–20.90	–12.97	–21.12	–21.23
*y*_122_	0.42	–0.41	0.42	0.45
*y*_222_	–0.93	–0.69	–0.93	–0.92
*y*_223_	0.12	0.18	0.18	0.27
*g*_22_	–4.58	–4.71	–4.59	–4.39

**Figure 3:**
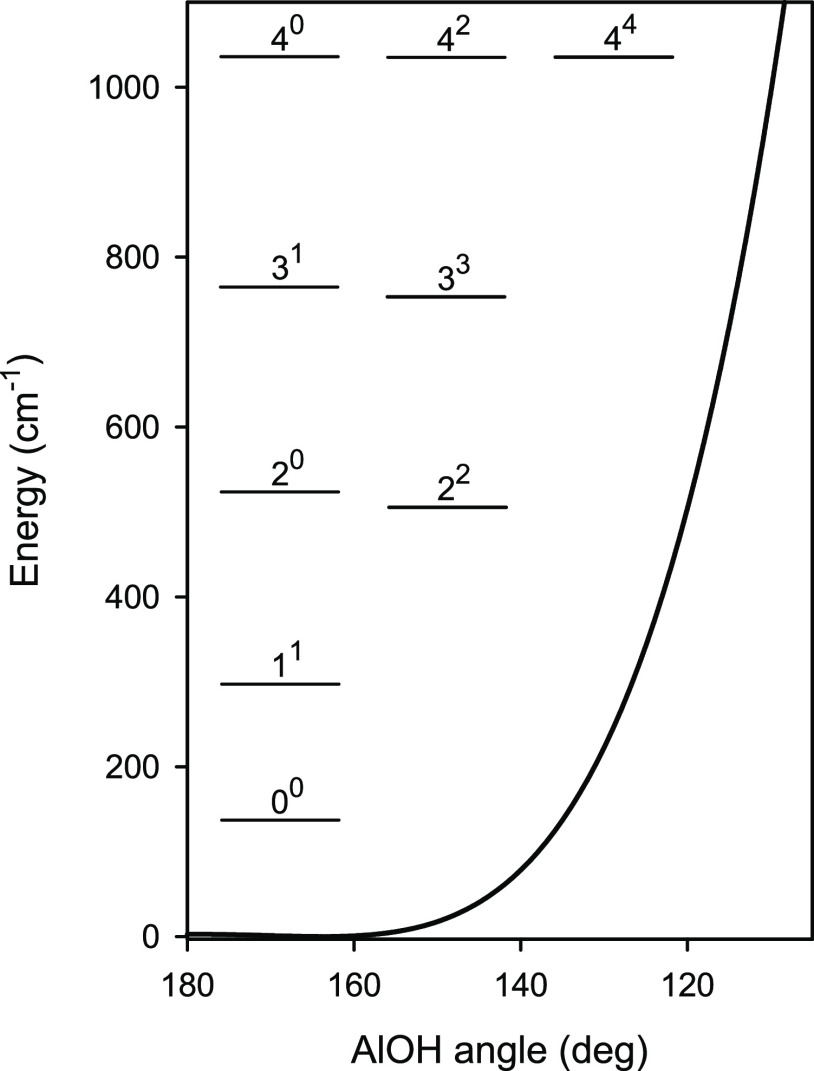
Minimum-energy potential function along the AlOH valence angle and the location of the ν_2_^*l*^ bending energy levels (*N* = |*l*|, ν_1_ = ν_3_ = 0) for the main isotopologue AlOH.

The predicted minimum-energy potential function along the AlOH valence angle is shown in Figure 3. Using the adiabatic potential energy surface *V*(*q*_1_, *q*_2_, θ) of the main isotopologue AlOH, the minimum-energy potential function *V*_mep_(θ) was determined to be
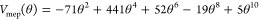
2where the potential energy *V* and the coordinate θ are given in wavenumbers and radians, respectively. The bending potential energy function of AlOH is thus essentially a quartic function, with a sizable negative quadratic contribution. The remarkable flatness of the bending potential energy function near a minimum is a reason why both the experimental and theoretical values of the equilibrium AlOH angle are rather uncertain. In such a case, the shape of the bending potential energy function becomes a crucial part of the structural definition of the AlOH molecule. The function *V*_mep_(θ) is fairly close to a one-dimensional slice through the potential energy surface *V*(*q*_1_ = 0, *q*_2_ = 0,θ). Figure 3 also shows the location of the ν_2_^*l*^ bending energy levels (*N* = |*l*|, ν_1_ = ν_3_ = 0) for the main isotopologue AlOH. The ground bending state of AlOH was calculated to lie 138 cm^–1^ in energy above the top of the barrier to linearity. The classical turning point for this state is located at the AlOH valence angle of 135°. The vibrational amplitude of the ν_2_ mode of the main isotopologue AlOH at any of its energy levels is thus larger than 45°. Clearly, the bending mode ν_2_ of AlOH can be called a large-amplitude motion.

**Figure 4:**
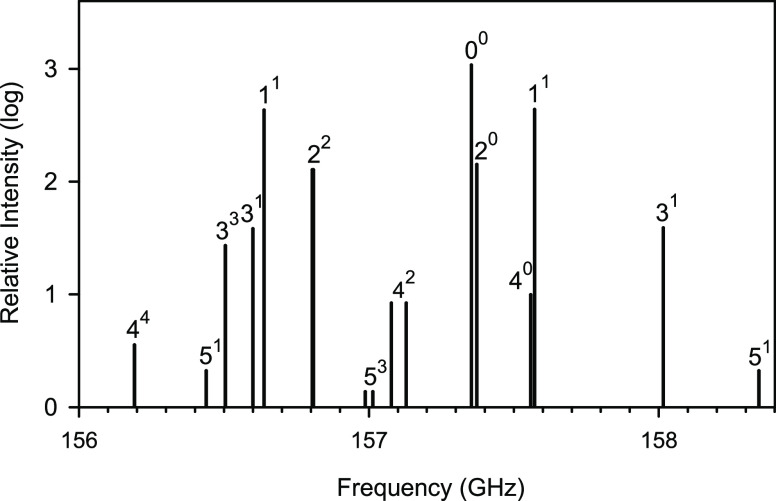
Stick diagram showing the *a*-type *N* = 5 ← 4 rotational transitions of the main isotopologue AlOH arising from molecules in various bending energy levels. The lines are labeled with the ν_2_^*l*^ quantum numbers. A logarithmic scale is used for the relative intensity.

The predicted effective rotational *B*_ν1_, ν_2_^*l*^, and ν_3_ and quartic centrifugal distortion *D*_ν1_, ν_2_^*l*^, and ν_3_ constants of the low-lying vibrational states of the AlOH, AlOD, ^26^AlOH, and Al^18^OH isotopologues are given in Table 5. The rotational spectra of AlOH and AlOD were observed by Apponi et al.,^3^ and the spectroscopic constants for the ground vibrational state were derived. For the AlOH isotopologue, the constants *B*_(0,0°,0)_ and *D*_(0,0°,0)_ were determined to be 15,740.3476 and 0.024812 MHz, respectively. For the AlOD isotopologue, the corresponding constants were determined to be 14,187.9524 and 0.019564 MHz, respectively. For both isotopologues, the constants *B*_(0,0°,0)_ and *D*_(0,0°,0)_ predicted in this work differ from their experimental counterparts by about 4 and 0.001 MHz, respectively. Table 5 also lists changes in the effective rotational constant due to excitation of the vibrational modes of AlOH: Δ*B* = *B*_(ν_1_,ν_2_^*l*^,ν_3_)_ – *B*_(0,0°,0)_. The predicted changes are expected to be accurate to ±1 MHz. Note that the changes Δ*B* due to excitation of the bending mode ν_2_ are quite large and irregular. To illustrate the pattern of rotational transitions in the low-lying excited ν_2_^*l*^ states, Figure 4 shows a part of the simulated *a*-type *N* = 5 ← 4 rotational spectrum of the main isotopomer AlOH. The relative line intensities at 298 K were roughly estimated using formulas for a linear molecule.^35^ At first sight, the predicted pattern of rotational transitions in the excited ν_2_^*l*^ states of AlOH appears to be somewhat chaotic. It resembles neither the regular pattern characteristic of a semirigid linear molecule nor that characteristic of a well-bent molecule. The most recognizable motif in this pattern is perhaps the series of the ν_2_^0^ lines (with ν_2_ = 0, 2, and 4) flanked more or less symmetrically by the (ν_2_ + 1)^1^*l*-type doublet lines. However, such a motif is characteristic of a semirigid triatomic molecule with the bent equilibrium configuration. In the *a*-type rotational spectrum of a well-bent molecule, the *k* = 0 lines for the ground and excited states of the bending mode are flanked almost symmetrically by the *k* = ± 1 asymmetry doublet lines (*k* here denotes the rotational quantum number, *k* = 0, ± 1, ···, ± *N*). The correspondence between the two motifs can be better understood by bearing in mind the relations between the quantum numbers: ν_2_ = 2*v*_b_ + |*k*| and *l* = *k*, where *v*_b_ denotes the bending quantum number of a well-bent molecule.

**Table 5:** Predicted Effective Rotational *B*_(ν_1_,ν_2_^*l*^,ν_3_)_ and Quartic Centrifugal Distortion *D*_(ν_1_,ν_2_^*l*^,ν_3_)_ Constants (in MHz) of the Low-Lying Vibrational States for the *X̃*^1^A′ State of AlOH, AlOD, ^26^AlOH, and Al^18^OH

	AlOH			AlOD			^26^AlOH			Al^18^OH		
ν_1_,ν_2_^*l*^,ν_3_	*B*	*D* × 10^3^	Δ*B*^a^	*B*	*D* × 10^3^	Δ*B*^a^	*B*	*D* × 10^3^	Δ*B*	*B*	*D* × 10^3^	Δ*B*^a^
0,0°,0	15,736.5	22.81	0.0	14,183.6	19.56	0.0	15,963.3	23.52	0.0	14,830.2	20.36	0.0
0,1^1*c*^,0	15,665.0	27.01	–71.5	14,169.4	18.75	–14.2	15,890.4	27.70	–72.9	14,756.3	18.39	–73.9
0,1^1*d*^,0	15,757.8	25.51	21.3	14,279.2	20.94	95.6	15,985.9	26.25	22.6	14,839.5	21.77	9.3
0,2^2*c*^,0	15,681.8	33.69	–54.7	14,247.8	33.26	64.2	15,908.1	34.94	–55.2	14,763.7	31.11	–66.5
0,2^2*d*^,0	15,681.8	25.11	–54.7	14,247.9	19.68	64.3	15,908.2	25.81	–55.1	14,763.7	23.55	–66.5
0,2°,0	15,737.7	9.60	1.2	14,268.5	7.22	84.9	15,965.2	9.81	1.9	14,818.4	12.98	–11.8
0,3^3^,0	15,651.5	31.61	–85.0	14,263.1	26.60	79.5	15,877.3	32.67	–86.0	14,730.1	27.96	–100.1
0,3^1*c*^,0	15,660.3	8.70	–76.2	14,218.9	12.22	35.3	15,886.0	9.07	–77.3	14,743.1	6.74	–87.1
0,3^1*d*^,0	15,802.4	17.64	65.9	14,385.4	15.26	201.8	16,032.1	18.08	68.8	14,870.1	15.54	39.9
0,4^4^,0	15,620.0	44.47	–116.5	14,272.4	28.24	88.8	15,845.2	45.60	–118.1	14,696.3	42.86	–133.9
0,4^2*c*^,0	15,712.1	105.26	–24.4	14,324.1	79.63	140.5	15,939.6	109.42	–23.7	14,783.2	96.39	–47.0
0,4^2*d*^,0	15,712.7	–1.49	–23.8	14,324.5	11.96	140.9	15,940.2	–1.50	–23.1	14,783.8	–3.96	–46.4
0,4°,0	15,751.5	–90.42	15.0	14,346.0	–46.83	162.4	15,979.9	–93.79	16.6	14,820.8	–84.81	–9.4
1,0°,0	15,606.6	24.80	–129.9	14,077.9	19.38	–105.7	15,830.6	25.53	–132.7	14,710.8	22.37	–119.4
0,0°,1	15,683.3	–52.06	–53.2	14,138.2	20.04	–45.4	15,909.2	–51.29	–54.1	14,776.9	–494.37	–53.3

a Changes in *B*_(ν_1_,ν_2_^*l*^,ν_3_)_ relative to the ground-state value.

To quantify the quasilinearity of the AlOH molecule, the parameter γ_0_ can be calculated.^36^ The parameter γ_0_ was defined to range from −1 for an ideal linear molecule to +1 for an ideal bent molecule. For the main isotopologue AlOH, the parameter γ_0_ is determined here to be −0.66. It is identical to that of fulminic acid—the prominent example of a quasilinear molecule^36^—and intermediate between those of magnesium and beryllium monohydroxides: γ_0_ = −0.74 and −0.15, respectively.^14,33^

## Conclusions

The equilibrium configuration of the AlOH molecule in its ground electronic state *X̃*^1^A′ was confirmed to be bent. The equilibrium AlOH angle was predicted to be 163°, and the barrier to linearity of the AlOH molecule was predicted to be just 4 cm^–1^. The vibration–rotation energy levels of the main AlOH molecule and its minor isotopologues were calculated to near “spectroscopic” accuracy. It was shown that the predicted pattern of rotational transitions in the ground and excited bending states of AlOH resembled neither the regular pattern characteristic of a semirigid linear molecule nor that characteristic of a well-bent molecule. The AlOH molecule was definitely concluded to be quasilinear. The theoretical results obtained in this work extend the spectroscopic knowledge of the AlOH molecule and will hopefully assist further experimental work, especially the high-resolution vibration–rotation spectroscopic studies.

## Supporting Information Available

Expansion coefficients *c_ijk_* of the Born–Oppenheimer potential energy surface V+C+H+R (au) (Table S1) (PDF)
