# Supplementary material for: Ab Initio Potential Energy Surface and Vibration–Rotation Energy Levels of Aluminum Monohydroxide
Source: J Phys Chem A. 2023 Oct 4;127(41):8607–14. doi: 10.1021/acs.jpca.3c05635 (PMC10591505; doi:10.1021/acs.jpca.3c05635)
Supplement: Supplementary file 1 — jp3c05635_si_001.pdf [file jp3c05635_si_001.pdf]

Ab Initio Potential Energy Surface and Vibration-Rotation Energy Levels of Aluminum Monohydroxide
Jacek Koput
Department of Chemistry, Adam Mickiewicz University, 61-614 Poznan, Poland

Table S1: The expansion coefficients c_{ijk} of the Born-Oppenheimer potential energy surface V+C+H+R (au)

i	*j*	*k*	*c*	*i*	*j*	*k*	*c*	*i*	*j*	*k*	*c*
0	0	2	-0.0003371500	2	0	0	1.4309437847	0	2	0	0.9287168780
0	0	4	0.0033218168	1	1	0	-0.0242409735	1	0	2	-0.0840707283
0	1	2	-0.0158478943	3	0	0	-1.5918359407	0	3	0	-0.1874555858
0	0	6	-0.0004331698	1	2	0	-0.0180335697	1	0	4	0.0220564513
0	1	4	-0.0019073459	2	1	0	-0.0626450882	2	0	2	0.0778284818
0	2	2	-0.0118200146	1	1	2	0.0171414729	4	0	0	-0.0033665344
0	4	0	-0.3438516986	0	0	8	0.0001116513	1	3	0	-0.0132681570
1	0	6	-0.0029687991	0	1	6	0.0000913053	3	1	0	0.0602071431
3	0	2	0.0122431423	0	3	2	0.0058537760	2	2	0	-0.0940731833
2	0	4	-0.0202634136	0	2	4	-0.0043621496	1	1	4	0.0084237047
1	2	2	-0.0201857336	2	1	2	-0.0363573414	5	0	0	0.7850863557
0	5	0	-0.3611282580	0	0	10	-0.0000090002	1	2	4	0.0305031316
2	1	4	0.0401220419	0	6	0	-0.3531794281	0	0	12	0.0000023085

r_{lin}(AlO)= 1.668068 Å r_{lin}(OH)= 0.948475 Å
